# Integrating suitability for teaching into an electronic health record - A novel and versatile tool for medical education

**DOI:** 10.15694/mep.2019.000126.1

**Published:** 2019-06-07

**Authors:** Himanshu Singh, Yvonne Thomson, Madhavi Paladugu, Nick Wood, Alexander Woywodt

**Affiliations:** 1 Lancashire Teaching Hospitals NHS Foundation Trust; 2 Lancashire Teaching Hospitals NHS Foundation Trust; 3 Lancashire Teaching Hospitals NHS Foundation Trust

**Keywords:** electronic health record, medical education, ward-based teaching

## Abstract

This article was migrated. The article was not marked as recommended.

The educational literature has noted the implications of electronic health records (EHR) for patient care and discussed various implications for the learner-teacher relationship but it has so far not viewed EHR as an educational tool. We wondered whether one could use EHR to facilitate undergraduate medical students’ exposure to hospital in-patients with an interesting history or findings on clinical examination. As clinicians, we encounter such patients on a regular basis during ward rounds and referrals but students are often absent during these encounters, leading to a loss of learning opportunities. Our aim was therefore to harness the EHR and create an electronic “flag” that would, following verbal consent, document suitable inpatients and thus maximise the students’ exposure to interesting findings on clinical examination. With help from our IT department we developed a simple add on to our existing EHR that allows any clinician to electronically highlight and document such patients during inpatient encounters. A member of the educational faculty can, whenever required, interrogate the EHR for the presence of inpatients with interesting findings on examination in the hospital and facilitate contact with our medical students. We report details of our approach, describe early experience and potential pitfalls and suggest future applications.

## Introduction

Medical Schools are currently training a generation of students for a workplace that will be dominated by electronic health records (EHR). The terms electronic medical record (EMR) and electronic health record (EHR) have been used interchangeably, although differences exist. The EMR is essentially a digital version of the paper charts created by one provider whereas the EHR ( Gunter and Terry, 2005) includes shared data from multiple providers. In contrast, a personal health record (PHR) is an application for recording personal medical data that is patient-controlled and made available to healthcare providers. The educational literature has described implications of EHR for quite some time now. Some medical professionals are sceptical ( Hammoud *et al.*, 2012b) or ambivalent about many of the changes brought about by EHRs ( Ellaway, Graves and Greene, 2013). Some authors have postulated competencies that Medical Schools should teach to prepare their students for the digital workplace of the future ( Hammoud *et al.*, 2012a). However to our best knowledge nobody has so far tried to modify EHR systems for educational purposes. In our practice in a large teaching hospital there are often patients, which we want to highlight to our undergraduate medical students, usually because they exhibit rare but important signs on clinical examination. Good examples include the murmur of aortic regurgitation, a vasculitic rash, or palpable splenomegaly. All three are important but reasonably rare in inpatients, difficult to appreciate without being in a room with the patient, and near impossible to teach with simulation or technology. In our clinical practice, we noted the increasing use of electronic “tags” on groups of patients for clinical purposes for example to highlight patients with renal impairment or diabetes. We wondered whether such electronic labels could be used for educational purposes.

## Methods and Results

### Study setting

Our hospital trust operates two hospital sites in the North West of England with 920 beds in total and most specialties on site with the exception of cardiothoracic surgery. It hosts around 280 medical students who are on a five year MbChB course at Manchester University and spend all of their three clinical years at our institution. The hospital trust operates a Harris Healthcare EHR system (Herndon VA/USA).The system in our institution fulfils the criteria for EHR as it includes access to laboratory results and imaging but also to primary care documentation via a Health information Exchange platform (Tiani GmbH, Vienna, Austria).

### Methods

**Figure 1.**
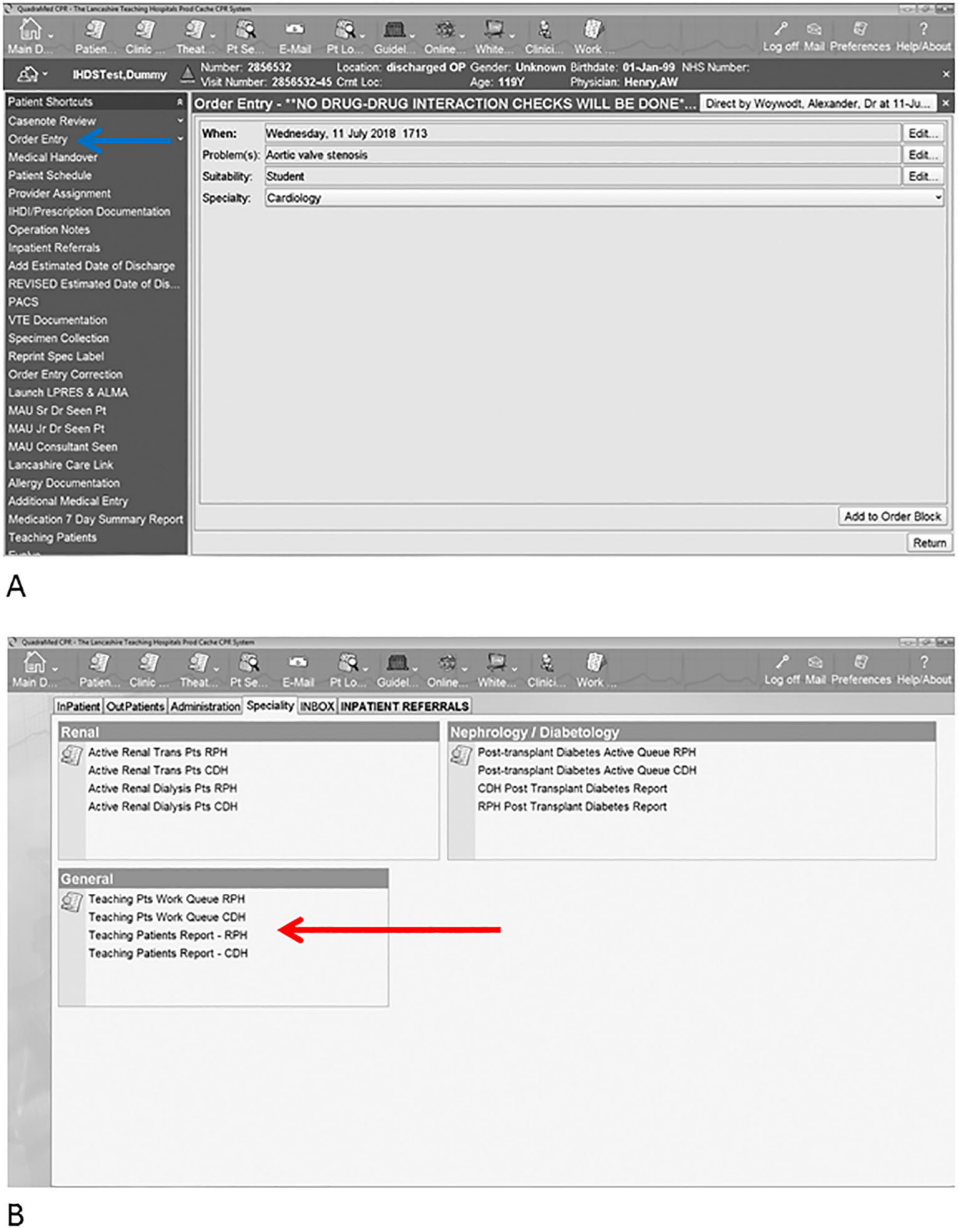
Use of the electronic label for teaching purposes on a fictitious patient. The user selects the patient and then selects the interesting clinical finding and the specialty from dropdown menus (in this case aortic stenosis and cardiology) (Panel A). The system then allows for lists to be generated for example by specialty or location (Panel B).

We first discussed the idea in spring 2016, liaised with our IT department and eventually submitted a change request to establish an additional “teaching label” on the EHR. We presented the request to the hospital trusts change board, which approved the request. The new facility on our EHR became functional as of September 2017. The “labelling” is easy to use ( Figure 1): Users choose “order” (Panel A, blue arrow) as if they would to order a laboratory test, imaging, or medication and then choose “Teaching Patient”. This will open up a drop down menu and the clinician can select the relevant specialty and the relevant finding from a range of options. The final step involves accepting the “order”. Of note, we ask for verbal consent (as we would have done in the past to gain consent to demonstrate findings to students) and we have also included a function to remove patients from the list if they so choose.

### Results

We limited our project to one senior clinician and one group of eight year 3 undergraduates during Academic year 2017/18. For convenience we chose our respiratory placement since one of the authors of this work is a respiratory physician. The Year 3 students had already been on the wards for initial induction and had been taught how to access the EHR. When they started in our Respiratory/Acute Medicine Firm, we delivered the timetable as usual for 3 weeks and then taught students how to access the HER Teaching List for the next 3 weeks. Normally the students would find out suitable patients for history taking and examination solely by asking ward staff. Clinicians found the system extremely user friendly and would typically complete the process in around 1-2 minutes, from taking verbal consent and documenting this in the notes to completing the process of EHR. Overall the student experience was very positive. In particular students commented in their verbal feedback that they were able to access more patients to their satisfaction and that the new approach greatly facilitated their exposure to interesting histories and findings on examination. Anecdotal feedback from patients, relatives and staff not involved in this project did not suggest any issues with this approach although as a team we had some concerns ourselves: Importantly, we were concerned about patients being overwhelmed by large numbers of students. This could not occur in this pilot study due to the limited numbers of students but may well be an issue if rolled out across the entire year 3 of just under 100 students. We suggest that some rules would be required, for example to limit access to patients by specialty so that for example the cardiology list (mainly patients with cardiac murmurs) would only be access by the cardiology placements supervisor and access limited to students on the cardiology placement. We also plan to limit access to placement supervisors and educational staff because the educational value of our approach can be further enhanced by a tutor guiding students to suitable patients or selecting presentations that contrast or complement each other.

Secondly, we were concerned that students would exchange information about interesting patients in between groups and therefore counteract our efforts to minimise crowding. We suggest that students are instructed to only access patients within their current placements specialty. Finally, we were concerned about consent and sought advice from our IT change board who took the view that consent should be taken in the usual way i.e. verbally and that this should be documented in the notes. Students were also asked to seek permission again verbally before seeing a patient.

## Discussion

The implications of EHR for training the future workforce have been discussed previously, including concerns that EHR may alter the interaction between teachers and learners. There is general consensus that the EHR is changing the current practice environment and that teaching practice needs to respond ( Elliott, Judd and McColl, 2011). Here, we describe how educators can actually use the EHR for medical education by electronically “labelling” those patients who are willing to participate in teaching and who have interesting findings on examination, or another feature of their disease that makes them particularly suitable for teaching. What our little report adds is a simple useful and versatile tool to harness EHR for educational purposes: Apart from the use described here we also see an opportunity for organising and hosting exams: It would be easy to widen the initial consent to include approval to be approached for exams and teaching after discharge. The electronic flag could be used to feed into a database of suitable patients for exams and postgraduate courses. There is also an opportunity here in rewarding patients for their participation in teaching: As an example, patients who have participated repeatedly could be sent a thank you letter and invited to participate in educational activities as expert patients. It could also be used to track cases over a longitudinal period and students could do case studies both in and out of hospital to enhance their understanding of the case.

This issue we address i.e. the mismatch of educationally rewarding cases and undergraduate student presence is becoming increasingly relevant for several reasons: Firstly, the turnover of patients is accelerating all the time with a national drive for outpatient management, so that patients with such findings will inevitably spend less time in hospital than they used to. Secondly, our students’ timetables are increasingly prescriptive with multiple commitments, such as skills training, group teaching, and communication training and students spend less time on wards than they used to. Thirdly, we as clinician educators are increasingly busy and lack the time to find students when presented with an interesting finding during ward rounds or when doing inpatient referrals.

Our approach has advantages, mainly the fact that it incurs very little cost (assuming that an in house IT department can facilitate the required software changes), requires very little or no training, and avoids an additional industry of databases or paper records of patients who are suitable for teaching. We emphasise the need for a user-friendly approach and we regarded the simplicity of our solution as a key to success: Clinicians are already used to ordering laboratory tests, imaging, or medication in the way described and all we have added is the “suitability for teaching” as an additional item on the order list.

We also acknowledge the limitations of our study. Our report lacks data on actual usage and student satisfaction. We have highlighted some potential issues with our approach above and suggested possible solutions which we will consider when rolling out our approach across the whole of our Year 3 undergraduate teaching. An additional minor issue we can envisage is the need to encourage and motivate already busy clinicians outside the core educational faculty to use this system and enrol patients during their busy clinical work. Our approach will be to advertise the new development and perhaps reward enthusiastic adopters in our existing system of teaching awards. We also recognise that our hospital’s IT infrastructure was key to the success of our project and that lack of access to IT resources or use of an off-the-shelf EHR may preclude the approach described here. Finally we acknowledge that our previous longstanding involvement in IT design and development in our institution helped us enormously to drive this project forward.

## Take Home Messages

Our early experience with an electronic flag for educationally rewarding patients has been very positive and we would like to encourage others to share our approach. In theory, and with help from a supportive IT department, every hospital with EHR should be able to implement our electronic flagging system for teaching purposes. Further work should evaluate the use of our approach in more detail, study perceptions of patients and relatives and consider more innovative ways to use EHR for educational purposes.

## Notes On Contributors

Himanshu Singh is a Consultant Respiratory Physician, Year 3 Placement Supervisor and Area Lead at Lancashire Teaching Hospitals NHS Foundation Trust and Associate Year 3 Lead at Manchester Medical School.

Yvonne Thomson is a Year 3 and Year 5 Clinical Placement Facilitator Lead at Lancashire Teaching Hospitals NHS Foundation Trust.

Madhavi Paladugu is a Consultant Paediatrician and Hospital Dean at Lancashire Teaching Hospitals NHS Foundation Trust.

Nick Wood is Consultant Gynaecologist and Chief Clinical Information Officer at Lancashire Teaching Hospitals NHS Foundation Trust.

Alexander Woywodt is a Consultant Nephrologist and Honorary Clinical Professor in Medicine and Associate Undergraduate Dean for Year 3 at Lancashire Teaching Hospitals NHS Foundation Trust.

HS came up with the idea and progressed it with the hospitals IT department with support from NW as Chief Clinical Information Officer and with support from AW regarding educational governance. HS trialled the approach on his Year 3 undergraduate placement. HS wrote the first concept of the manuscript. AW worked on the manuscript from concept stage to submission with regular input from HS. YT and PM supported the project and provided helpful discussion and comment.

All authors have seen and approved the final version of the manuscript.
