# Integrating suitability for teaching into an electronic health record - A novel and versatile tool for medical education

**DOI:** 10.15694/mep.2019.000126.2

**Published:** 2020-03-23

**Authors:** Himanshu Singh, Yvonne Thomson, Madhavi Paladugu, Nick Wood, Alexander Woywodt

**Affiliations:** 1 Lancashire Teaching Hospitals NHS Foundation Trust; 2 Lancashire Teaching Hospitals NHS Foundation Trust; 3 Lancashire Teaching Hospitals NHS Foundation Trust; 4 Lancashire Teaching Hospitals NHS Foundation Trust; 5 Lancashire Teaching Hospitals NHS Foundation Trust; 6 Lancashire Teaching Hospitals NHS Foundation Trust

**Keywords:** electronic health record, medical education, ward-based teaching

## Abstract

This article was migrated. The article was marked as recommended.

The educational literature has noted the implications of electronic health records (EHR) for patient care and discussed various implications for the learner-teacher relationship but it has so far not viewed EHR as an educational tool. We wondered whether we could use EHR to facilitate undergraduate medical students’ exposure to hospital in-patients with an interesting history or findings on clinical examination. As clinicians, we encounter such patients on a regular basis during ward rounds and referrals but students are often absent during these encounters, leading to a loss of learning opportunities. Our aim was therefore to harness the EHR and create an electronic “flag” that would, following consent, document suitable inpatients and thus maximise the students’ exposure to patients who present learning opportunities. With help from our IT department we developed a simple add on to our existing EHR that allows any clinician to electronically highlight and document such patients during inpatient encounters. A member of the educational faculty can, whenever required, interrogate the EHR for the presence of inpatients with interesting findings on examination in the hospital and facilitate contact with our medical students. We report details of our approach, describe early experience and potential pitfalls and suggest future applications.

## Introduction

Medical Schools are currently training a generation of students for a workplace that will be dominated by electronic health records (EHR). The terms electronic medical record (EMR) and electronic health record (EHR) have been used interchangeably, although differences exist. The EMR is essentially a digital version of the paper charts created by one provider whereas the EHR ( Gunter and Terry, 2005) includes shared data from multiple providers. In contrast, a personal health record (PHR) is an application for recording personal medical data that is patient-controlled and made available to healthcare providers.

The educational literature has described implications of EHR for quite some time now. Some medical professionals are sceptical ( Hammoud *et al.*, 2012b) or ambivalent about many of the changes brought about by EHRs ( Ellaway, Graves and Greene, 2013). Some authors have postulated competencies that Medical Schools should teach to prepare their students for the digital workplace of the future ( Hammoud *et al.*, 2012a). Others have studied how undergraduate medical students access and use EHR and raised the issue of how much time students should actually spend on computers ( Chi *et al.*, 2014). While many EHR systems allow for student login and other options, such as writing notes, there is limited data on how to modify EHR systems for educational purposes although Elliott and co-workers reported early experience with a student-centred EHR ( Elliott, Judd and McColl, 2011).

In our practice in a large teaching hospital there are often patients, which we want to highlight to our undergraduate medical students, for example because of an interesting history or because they exhibit rare but important signs on clinical examination. Good examples include the murmur of aortic regurgitation, a vasculitic rash, or palpable splenomegaly. All three are important but reasonably rare in inpatients, difficult to appreciate without being in a room with the patient, and near impossible to teach with simulation or technology. In our clinical practice, we noted the increasing use of electronic “tags” on groups of patients for clinical purposes for example to highlight patients with renal impairment, allergies or diabetes. We wondered whether such electronic labels could be used for educational purposes.

## Methods and Results

### Study setting

Our hospital trust operates two hospital sites in the North West of England with 920 beds in total and most specialties on site with the exception of cardiothoracic surgery. It hosts around 280 medical students who are on a five year MbChB course with Manchester University and spend all of their three clinical years at our institution. The hospital trust operates a QuadraMed™ EHR system (Quadramed Corp., Plano/Tx, USA). The system in our institution fulfils the criteria for EHR as it includes access to laboratory results and imaging but also to primary care documentation via a Health information Exchange platform (Tiani GmbH, Vienna, Austria).

### Methods

**Figure 1.**
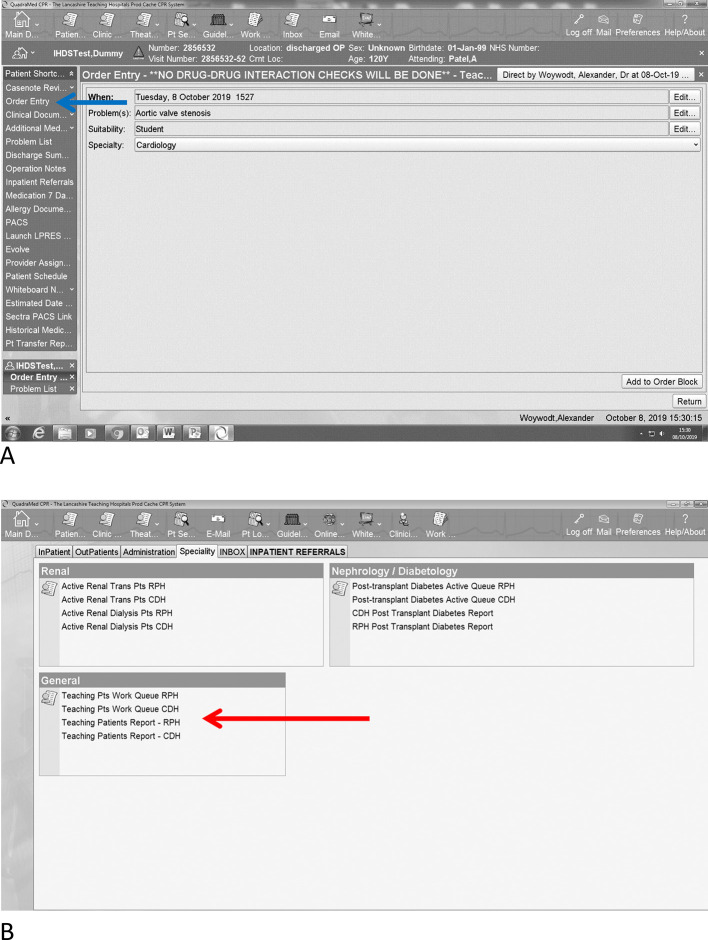
Use of the electronic label for teaching purposes on a fictitious patient. The user selects the patient and then selects the interesting clinical finding and the specialty from dropdown menus (in this case aortic stenosis and cardiology) (Panel A). The system then allows for lists to be generated for example by specialty or location (Panel B).

We first discussed the idea in spring 2016, liaised with our IT department and eventually submitted a change request to establish an additional “teaching label” on the EHR. We presented the request to the hospital trusts change board, which approved the request. The new facility on our EHR became functional as of September 2017. The “labelling” is easy to use ( Figure 1): Users choose “order” (Panel A, blue arrow) as if they would to order a laboratory test, imaging, or medication and then choose “Teaching Patient”. This will open up a drop down menu and the clinician can select the relevant specialty and the relevant finding from a range of options. The final step involves accepting the “order”. Of note, we ask for verbal consent (as we would have done in the past to gain consent to demonstrate findings to students) and we have also included a function to remove patients from the list if they so choose. We also carried out a very brief paper based survey among one group of Year 3 students in academic year 2017/18 (n=8).

## Results

We limited our project to one senior clinician and one group of eight year 3 undergraduates during Academic year 2017/18. For convenience we chose our respiratory placement since one of the authors of this work is a respiratory physician. The Year 3 students had already been on the wards for initial induction and had been taught how to access the EHR. When they started in our Respiratory/Acute Medicine placement, we delivered the timetable as usual for 3 weeks and then taught students how to access the EHR patient list for the next 3 weeks.

Normally the students would find suitable patients for history taking and examination solely by asking ward staff. Clinicians found the system extremely user-friendly and would typically complete the process of “labelling” in around 1-2 minutes, from taking verbal consent and documenting this in the notes to completing the process. Overall the student experience was very positive. In particular students commented in their verbal feedback that they were able to access more patients to their satisfaction and that the new approach greatly facilitated their exposure to interesting histories and findings on examination. In a brief survey of student satisfaction most students agreed or agreed strongly with the utility of our new approach ( Table 1). Some lower scores were received from some students with regards to ease of use for identification of patients (Q3) and for tracking (Q6).

Anecdotal feedback from patients, relatives and staff not involved in this project did not suggest any issues with this approach although as a team we had some concerns ourselves: Importantly, we were concerned about patients being overwhelmed by large numbers of students. We were also considered the possibility that students would exchange information about interesting patients in between groups and therefore counteract our efforts to minimise crowding. Neither did occur in our pilot, presumably due to the relatively small number of students involved but may well be an issue if rolled out across the entire year 3 of just under 100 students.

**Table 1.** Paper based survey of student satisfaction in one group of Year 3 students in Academic Year 2017/18 (n=8)

	Strongly disagree	Disagree	Neutral	Agree	Strongly Agree
Q 1: It was easy to find appropriate patients for history and examination on the ward	0	0	1	4	3
Q 2: Overall, the use of the QCPR teaching list to find new patients on the wards is easy for day to day learning	0	0	0	2	6
Q 3: Overall, the use of the QCPR teaching list to highlight particular speciality/disease patients on wards for history and examination is easy for day to day learning	0	1	2	1	4
Q 4: Overall the Teaching List on QCPR allows efficient use of time in finding patients and you could use the time for other learning tasks vs medical team/board system	0	0	0	2	6
Q 5: I am really satisfied with the QCPR Teaching list help in finding appropriate students on the wards	0	0	1	2	4
Q 6: It is easy to track and follow the particular system/pathology patients on ward with the use of the teaching list on QCPR	1	0	3	2	2
Q 7: The patients highlighted by medical team on the QCPR teaching list were appropriate for your learning?	0	0	0	1	7
Q 8: The patients you’ve seen through the QCPR teaching list, with regards to their clinical problems were quite varied on the ward	0	0	2	5	1

## Discussion

The implications of EHR for training the future workforce have been discussed previously, including concerns that EHR may alter the interaction between teachers and learners. There is however widespread consensus that the EHR is changing the clinical environment and that educational practice needs to respond ( Elliott, Judd and McColl, 2011). Here, we describe how educators can actually use the EHR for medical education by electronically “labelling” those patients who are willing to participate in teaching and who have an interesting history or interesting findings on examination to make them particularly suitable for teaching.

What our little report adds is a simple useful and versatile tool to harness EHR for educational purposes: Apart from the use described here we also see an opportunity for organising and hosting exams: It would be easy to widen the initial consent to include approval to be approached for exams and teaching after discharge. The electronic flag could be used to feed into a database of suitable patients for exams and postgraduate courses. There is also an opportunity here in rewarding patients for their participation in teaching: As an example, patients who have participated repeatedly could be sent a thank you letter and invited to participate in educational activities as expert patients. It could also be used to track cases over a longitudinal period and students could do case studies both in and out of hospital to enhance their understanding of the case.

This issue we address i.e. the mismatch of educationally rewarding cases and undergraduate student presence is becoming increasingly relevant for several reasons: Firstly, the turnover of patients is accelerating all the time with a national drive for outpatient management, so that patients with such findings will inevitably spend less time in hospital than they used to. Secondly, our students’ timetables are increasingly prescriptive with multiple commitments, such as skills training, group teaching, and communication training and students spend less time on wards than they used to. There is also pressure to accommodate more learners generally (Melvin *et al.*). Thirdly, we as clinician educators are increasingly busy and lack the time to find students when presented with an interesting finding during ward rounds or when doing inpatient referrals.

Our approach has advantages, mainly the fact that it incurs very little cost (assuming that an in house IT department can facilitate the required software changes), requires very little or no training, and avoids an additional industry of databases or paper records of patients who are suitable for teaching. We emphasise the need for a user-friendly approach and we regarded the simplicity of our solution as a key to success: Clinicians are already used to ordering laboratory tests, imaging, or medication in the way described and all we have added is the “suitability for teaching” as an additional item on the order list.

We also acknowledge the limitations of our study. Our report lacks data on actual usage and student satisfaction. One key issue we envisage is that of crowding caused by student demand for unusual findings in a given patient. We have identified several options to address this when we roll out this approach to the entire Year 3 with just under 100 students. Firstly, we are planning to limit access to placement supervisors and educational staff as opposed to giving students direct access to the database. This will also likely enhance the educational value of our approach by a tutor guiding students to suitable patients or selecting presentations that contrast or complement each other (for example highlighting a patient with mitral stenosis to a group who has just seen a patient with mitral regurgitation). An additional challenge this would address is to find an appropriate balance between common and rare clinical scenarios. In this regard the tutor could ensure that such a balance is maintained whereas if the students had direct access to the database they may focus on more unusual scenarios. It may also be necessary to limit students’ access by area, so that for example a patient with cardiac murmur is only accessible to students on our cardiology and respiratory Year 3 placements as opposed to the entire cohort. A final option would be to incorporate an electronic “counter” in the system that counts student encounters with a given patient and flags once a specified number of encounters have been reached.

It is worth reflecting on wider implications of our approach. Others have emphasised that technology should be regarded as a matter of ethics and professionalism and not solely as one of instrumentation ( Ellaway, 2014). Bardach and colleagues note that use of EHR can affect inter-professional communication, often in subtle ways ( Bardach, Real and Bardach, 2017). In this regard directing students to patients by means of technology may ever so slightly dis-incentivise them to for example speak to the ward manager or trainee doctors to find suitable patients in the clinical workplace. It is also possible that our approach may affect the willingness of patients to interact with students: Patients may be more willing to spend time with learners if they have been introduced by a clinician with whom they have a longstanding professional relationship and they may be sceptical or less willing if the contact is facilitated by the EHR. In our further evaluation of this approach we are planning to incorporate feedback from patients, relatives and ward staff to seek for evidence along these lines.

We were also slightly concerned about consent and sought advice from our IT change board who took the view that consent should be taken in the usual way i.e. verbally and that this should be documented in the notes. Students were also asked to seek permission again verbally before seeing a patient. Our survey of student satisfaction ( Table 1) also suggests that ease of use and utility for patient tracking can be improved.

An additional minor issue we can envisage is the need to encourage and motivate already busy clinicians outside the core educational faculty to use this system and enrol patients during their busy clinical work. An element of technology fatigue and reduced enthusiasm caused by increased use of EHR for teaching has been described previously ( Spencer *et al.*, 2012). Our approach will be to advertise the new development and perhaps reward enthusiastic adopters in our existing system of teaching awards. We also recognise that our hospital’s IT infrastructure was key to the success of our project and that lack of access to IT resources or use of an off-the-shelf EHR may preclude the approach described here. Finally we acknowledge that our previous longstanding involvement in IT design and development in our institution helped us enormously to drive this project forward.

## Take Home Messages

Our early experience with an electronic flag for educationally rewarding patients has been very positive and we would like to encourage others to share our approach. In theory, and with help from a supportive IT department, every hospital with EHR should be able to implement our electronic flagging system for teaching purposes. Further work should evaluate the use of our approach in more detail, study perceptions of patients and relatives and consider more innovative ways to use EHR for educational purposes.

## Notes On Contributors

Himanshu Singh is a Consultant Respiratory Physician, Year 3 Placement Supervisor and Area Lead at Lancashire Teaching Hospitals NHS Foundation Trust and Associate Year 3 Lead at Manchester Medical School.

Yvonne Thomson was at the time of writing a Year 3 and Year 5 Clinical Placement Facilitator Lead at Lancashire Teaching Hospitals NHS Foundation Trust. She is now a Multi Disciplinary Workforce Coordinator at West Lancashire CCG in Ormskirk, UK.

Madhavi Paladugu is a Consultant Paediatrician and Hospital Dean at Lancashire Teaching Hospitals NHS Foundation Trust.

Nick Wood is Consultant Gynaecologist and Chief Clinical Information Officer at Lancashire Teaching Hospitals NHS Foundation Trust.

Alexander Woywodt is a Consultant Nephrologist and Honorary Clinical Professor in Medicine and Associate Undergraduate Dean for Year 3 at Lancashire Teaching Hospitals NHS Foundation Trust.

HS came up with the idea and progressed it with the hospitals IT department with support from NW as Chief Clinical Information Officer and with support from AW regarding educational governance. HS trialled the approach on his Year 3 undergraduate placement. HS wrote the first concept of the manuscript. AW worked on the manuscript from concept stage to submission with regular input from HS. YT and PM supported the project and provided helpful discussion and comment. AW addressed reviewer comments during the revision process.

All authors have seen and approved the final version of the manuscript.
