# Supplementary material for: Integrating suitability for teaching into an electronic health record - A novel and versatile tool for medical education
Source: MedEdPublish (2016). 2020 Mar 23;8:126. Originally published 2019 Jun 7. [Version 2] doi: 10.15694/mep.2019.000126.2 (PMC10712509; doi:10.15694/mep.2019.000126.2)
Supplement: Peer review history

# Reviewer report: Reviewer response for version 2

**DOI:** 10.21956/mep.20247.r31404

**Published:** 2020-03-28

**Authors:** Madalena Folque Patricio

**Affiliations:** 1 School of Medicine, University of Lisbon

This review has been migrated. The reviewer awarded 5 stars out of 5 Before going ahead, let me say that I have read the Version 2 of this article (not the initial version) where, according to the authors, the changes suggested by previous reviewers were introduced. They are all very pertinent. The authors should be congratulated for this interesting article. Their proposal addresses the lack of ‘systematization’ when teaching occurs in clinical settings, depending only in patients’ availability. This problem, concerning the lack of systematic clinical teaching opportunities, was highlighted already in 1984 by Ronald Harden. In his article Educational Strategies in curriculum development: The SPICES Curriculum, he argues that ‘teaching cannot continue to be left to chance’, i.e. teaching cannot be left to the opportunities that arise in the wards.The use of electronic health records (EHR) to identify good opportunities for clinical teaching is contributing to more ‘systematization’ in clinical settings. According to the authors, good potential opportunities for teaching can easily be identified and planned, avoiding the risk of being lost. In fact, the referencing system allows easy identification of relevant pathologies, facilitating the planning of teaching.I especially appreciated the authors' concern to introduce a system that simultaneously protects the patient from an excessive number of students. This is not only a very important aspect for the patients’ well-being but also for students’ awareness that patients’ well-being must be a priority.Other positive aspects concern the easy transference of this proposal to other educational scenarios and the fact that cost is irrelevant, according to what authors said.There are however two aspects needing attention. One concerns the fact that the use of EHR was tested with only 1 doctor and 8 students. The authors are aware of this limitation, which requires more attention if implementation is to be made with a large number of students. The second relates to the results of Survey Question 6, namely ‘It is easy to track and follow the particular system/pathology patients on ward with the use of the teaching list on QCPR’, which indicate some problems. These problems need clarification because although the authors are aware, they contradict what was reported in terms of easy implementation of the system To conclude, I would say again that this is an interesting article that deserves our reading.

Reviewer Expertise:

NA

No decision status is available

# Reviewer report: Reviewer response for version 2

**DOI:** 10.21956/mep.20247.r31401

**Published:** 2020-03-26

**Authors:** Barbara Jennings

**Affiliations:** 1 Norwich Medical School

This review has been migrated. The reviewer awarded 4 stars out of 5 Thank you very much for this revised version that has thoroughly addressed the reviewers’ questions and suggestions.Thank you also for a very interesting and well-presented article that presents the use of efficient teaching opportunities linked to the electronic health record. This is an excellent idea for a workplace based educational tool. I think this article will be of broad interest to all colleagues who deliver clinical teaching.

Reviewer Expertise:

NA

No decision status is available

# Reviewer report: Reviewer response for version 2

**DOI:** 10.21956/mep.20247.r31400

**Published:** 2020-03-26

**Authors:** Subha Ramani

**Affiliations:** 1 Harvard Medical School, Brigham and Women's Hospital

This review has been migrated. The reviewer awarded 5 stars out of 5 I read this paper with great interest, being a clinician educator who conducts bedside rounds sometimes purely for teaching purposes. It is very difficult to identify patients for teaching sessions, given the immense volume of traffic (patients, learners, teachers, other staff) as well as ultra short inpatient stays.Clinicians are more often frustrated by switching to EHR practice as it comes with many challenges including increased documentation requirements and information overload. However, we do not often sit down and think about how this information can aid teaching. The authors have done a great job in recommending the use EHR for educational purposes.Methodology is appropriate, writing is very good and recommendations are very useful.They rightly point out the importance of information protection.One point I would like to emphasize is that patients should be informed that their diagnoses will be identifiable and may be used for such teaching sessions at teaching hospitals. we should also avoid crowds of learners coming to talk to or examine the one patient with an interesting diagnosis.This paper is a good start to the discussion and I look forward to further inquiry into this area and recommendations for clinical teaching.

Reviewer Expertise:

NA

No decision status is available

# Reviewer report: Reviewer response for version 2

**DOI:** 10.21956/mep.20247.r31402

**Published:** 2020-03-24

**Authors:** Trudie Roberts

**Affiliations:** 1 Leeds Institute of Medical Education

This review has been migrated. The reviewer awarded 4 stars out of 5 As EHRs become ubiquitous it is important that their value as educational tools is recognised and utilised and this paper is a good start in doing this.The discussion is particularly useful in setting out the potential obstacles and how the authors tried to address these issues. As the previous reviewer says the test now will be to scale up this for all students and trainees

Reviewer Expertise:

NA

No decision status is available

# Reviewer report: Reviewer response for version 2

**DOI:** 10.21956/mep.20247.r31403

**Published:** 2020-03-24

**Authors:** Ken Masters

**Affiliations:** 1 Sultan Qaboos University

This review has been migrated. The reviewer awarded 5 stars out of 5 The authors have done very well to address the reviewers’ concerns, and have gone further by expanding their Discussion with a broader reflection of practical problems and possible solutions that may confront them as they roll out the system to more students and staff. I look forward to seeing an updated paper detailing the research on a wider scale. Even at this stage, though, I am sure that many readers of this paper will ask their IT support “Why don’t _we_ have something like this?” Nicely done.

Reviewer Expertise:

NA

No decision status is available

# Reviewer report: Reviewer response for version 1

**DOI:** 10.21956/mep.18994.r27236

**Published:** 2019-10-15

**Authors:** Barbara Jennings

**Affiliations:** 1 Norwich Medical School

This review has been migrated. The reviewer awarded 3 stars out of 5 This is a very interesting article that introduces us to a new educational tool and an approach that has been designed to support undergraduate clinical teaching and learning opportunities. The potential for making the clinical and educational interface more efficient is striking. This approach could also be valuable for clinician CPD in critically important scenarios – such as the identification of rare adverse drug reactions.The rationale for using alerts and tags in electronic health records (EHR) and how the Health Information Exchange Platform can be configured for this task are outlined clearly. The screen shots presented in the methods section are useful illustrations. The integrated nature of the platform that was used in the secondary care setting (with access to laboratory and imaging data as well as primary care records) makes it particularly valuable for this educational application.The authors also carefully describe the information governance and ethical considerations associated with the development of an educational tool from the EHR, and the roles of each author in the project.I have a couple of suggestions and questions for the authors to consider in a revised manuscript, or in further research about the utility of this tool.1. A more detailed literature review of the validity of this type of educational activity, and the place of EHRs (in undergraduate and postgraduate medical education and assessment) could be useful for the reader.2. Giving undergraduate students a balanced perspective of both common clinical presentations, and rare but important scenarios is a challenge. Do the authors have any suggestions about this with respect to this EHR tool?3. A minor point, I noticed a few typos in the results section of the article.Although this article only presents a preliminary evaluation of the new tool, I think it will be of broad interest to colleagues who deliver clinical teaching - and it is well written and presented.

Reviewer Expertise:

NA

No decision status is available

# Reviewer report: Reviewer response for version 1

**DOI:** 10.21956/mep.18994.r27238

**Published:** 2019-09-30

**Authors:** Ken Masters

**Affiliations:** 1 Sultan Qaboos University

This review has been migrated. The reviewer awarded 3 stars out of 5 An interesting paper dealing with integrating EHRs into teaching.Some issues need addressing:• “However to our best knowledge nobody has so far tried to modify EHR systems for educational purposes.” This is a little misleading as many EMR and HER systems do allow areas for student input, and then the organisation can use that input as part of the teaching (e.g. for grand rounds, seminars, etc.) So, unless the authors are using “modify” in a particularly narrow and specifically-defined sense, they may wish to alter or qualify this statement somewhat. (In addition, there is at least one EMR system that is specifically designed for training medical students, so it might be useful to at least refer to that).• There was a concern about students’ exchanging information. In response, the authors provide a suggestion, but do not actually explain how they attempted to deal with this issue (and the lack of any evaluation (discussed below) does also leave the reader wondering about the effectiveness of the suggestion.).• Some of the information in Methods (e.g. suggestions and plans) should be moved to later in the paper.• Although the authors do note the lack of evaluation as a limitation, it really does severely limit the value of the paper. The authors really should have conducted even a basic evaluation to get some sense of the value, otherwise the paper remains in the “nice idea, perhaps” bracket. Perhaps for Version 2 of the paper, the authors will have had the chance to perform some form of rudimentary evaluation.Minor issues: Some of the paragraphs are really dauntingly long, and could benefit from being broken into smaller pieces.So, an interesting concept, but it would benefit from some more background clarity and some evaluation to get a sense of the viability of the project.

Reviewer Expertise:

NA

No decision status is available

# Reviewer report: Reviewer response for version 1

**DOI:** 10.21956/mep.18994.r27237

**Published:** 2019-06-10

**Authors:** Mary Higgins

**Affiliations:** 1 University College Dublin, National Maternity Hospital

This review has been migrated. The reviewer awarded 3 stars out of 5 This is a very interesting concept and thank you for sharing your pilot. I would be grateful if you could clarify how the EHR could log how many students have accessed each patient (as suggested in your note "patients who participate repeatedly" or does that mean on multiple admissions?When you roll this out are you planning on including patient feedback?

Reviewer Expertise:

NA

No decision status is available
